# Hospital Policy during the War

**Published:** 1915-01-23

**Authors:** 


### HOSPITAL POLICY DURING THE WAR.

The past four months of war have now consolidated the more ostensible part played by the voluntary hospitals in the reception and accommodation of the wounded. The lessons of experience now dictate and control the details and arrangement of the arrivals of the convoys, to some extent even the treatment of cases, since the average type of these is now known, and the dispatch of convalescents. All these points have been brought out by our special correspondents in the accounts which we have published during the four months. There is, therefore, no need to report the record of these matters week by week any longer. It is the problems still ahead with which we have to deal.

In this connection, therefore, we commend to the consideration of our readers the quotations from the report drafted by the house committee of the Halifax Royal Infirmary with reference to the problems arising out of the continued presence of wounded in the wards, economical and political, if we may use the last adjective to express the joint duty of the hospitals both to the civil and military class of patients, which we print in our Notes this week. It will be observed that the report was drawn up in answer to certain complaints made at the December meeting of the Insurance Committee, and in particular to answer the allegation that civil or insured patients were being displaced for the benefit of the wounded. All who read this very interesting document, as the quotations that we give sufficiently show, will be grateful that the report has been published, which would probably not have occurred if it had not been a *pièce d'occasion*, to answer criticism. It lays down clearly what is the policy for itself during the war, by showing that though voluntary hospitals were invited to co-operate with the military authorities, the strain on the latter's institutions in the early days was so great that had the hospitals refused (which, of course, they did not desire) they would probably have been commandeered. The question of accommodation, therefore, is still one to be solved at many institutions, since improvised hospital quarters and an often relatively inexperienced staff cannot be offered to serious cases among the wounded so long as hospital accommodation, in the technical sense, is anywhere available; and so long as the military hospitals need greatly supplementing.

We mention these points here to show why it is that any hospital war news that we may publish in the future must deal not so much with the now well-known details of detraining and arrival, but rather with the problems ahead, of which the above indicate the direction and scope. Will, therefore, those institutional correspondents who grasp what a fine opportunity now exists for intelligent hospital managers to extend the influence of the voluntary hospitals through the lively national interest at present generally manifested in hospital affairs, enter on the new phase of correspondence thus necessitated? The kind of matter it is helpful to publish is that contained in the Halifax Report; the kind of questions to be discussed are those ahead, and such as have only now begun to press; for instance, the problem created in certain hospitals by the prolonged stay of some of the wounded. Incidents of the work, the more remarkable medical cases; above all, a monthly report on the administrative and financial problems created by the new "sick and wounded department" and the new features introduced by this volume of special work: these are the points to be taken up and dealt with intelligently. If our special correspondents will help us in this matter, preferring a detailed and considered statement once a fortnight or once a month to the more frequent news paragraphs hitherto necessitated, we shall be able to place such a body of interesting evidence before the public as each individual hospital will benefit by, and by which the voluntary principle will be more firmly established in the public confidence than ever. To do this some care and discrimination are required by writers who realise the full scope of their present opportunity.

